# Self-reported unintentional injuries among youth (15–24 years) from Peshawar Pakistan: A cross-sectional survey on youth working in vocation institutes

**DOI:** 10.21203/rs.3.rs-2603081/v1

**Published:** 2023-03-09

**Authors:** Sarwat Masud, Adnan Hyder, Uzma Rahim Khan, Nadeem Ullah Khan, Ahmed Raheem, Pammla Petrucka

**Affiliations:** Aga Khan University Hospital; George Washington University; Aga Khan University Hospital; Aga Khan University Hospital; Aga Khan University Hospital; University of Saskatchewan

**Keywords:** Unintentional injuries, UIs, Pakistan, Youth, Adolescents, Peshawar

## Abstract

**Background:**

The burden of unintentional injuries among youth 15–24 years is high. There is paucity of data on unintentional injuries in youth working in Vocational Training Institutes.

**Objective:**

To determine the incidence, characteristics, and risk factors of non-fatal self-reported unintentional injuries among youth.

**Methods:**

**Design:**

This was a retrospective cross-sectional survey of youth recruited from vocational training centers in Peshawar Pakistan between February 2022 to October 2022.

**Participants:**

There were 547 study participants with 356 males and 191 females. Data were collected on self-reported unintentional injuries in the previous 12 months, using the WHO tool for injuries and violence [1]. Analysis for total injuries as an outcome and sociodemographic risk factors was done using a multilevel negative binomial regression model, adjusting for age and sex.

**Results:**

We documented a total of 503 injuries, with road traffic injuries being the most common (n=197, 39%), followed by burns (n=89, 18%) and falls (n=79,16%). Poisonings n=13%) and drownings (n=23, 7.1%) were the least common injuries. In-training occupational injuries reported were (n=95,18%). Females had a higher incidence rate of burns 2.19 [1.785–3.46] compared to males, while males had a higher incidence of RTI 3.24[2.35–5.3], falls 1.30 [0.74–2.27], poisonings 2.14 [0.57–7.58] and drownings 2.46(0.84–7.21).

lack of education 4.6 [1.12–18.91] (*p*=0.034), lack of helmet use 4.54 [2.12–9.76] (*p*=<0.001), lack of seat belt use 1.3 [1.14–1.69] (*p*= <0.001), smoking 1.25 [1.05–2.69] (*p*=0.049), lack of fathers education 4.71 [2.12–10.49] (*p*=<0.001), carrying a gun 6.59 [2.54–17.11] (*p*=<0.001), crowded families 3.59 [3.11–5.07] (*p*=<0.001) and lower family income 2.04 [1.04–4.02](*p*=0.039*) were significant risk factors for unintentional injuries in youth.

**Conclusion Added value of the study:**

This study provides up-to-date counts and incidence rates of unintentional injuries in youth from Peshawar Pakistan. It also provides a recent trend of the types of unintentional injuries among youth with sex-adjusted rates. The area of concern remains an increasing number of RTIs in both males and females and a higher rate of burn among females compared to males and falls among youth. A high incidence of in-training occupational injuries was reported among the vocational youth.

## Introduction

Unintentional injuries are a leading cause of death among youth 10–24 years old [2]. Globally, in 2019, there were 1·49 million deaths (1·39–1·59) in youth, with 61% of deaths occurring in males. Nearly one-third (32·7%) of the above-mentioned deaths were due to transport injuries, unintentional injuries, or interpersonal violence and conflict. Although deaths in this age group have decreased by 30% in females and 15·3% in males since 1950, the sex-based differences in mortality rate has widened in most regions of the world [3]. The World Health Organization confirms road traffic injuries RTI, drowning, and violence as major contributors youth mortality [4]. The Global Burden of Disease (GBD) Report in 2013 identified transport injuries (18·3% [95% UI 16·6–19·7]) as a major cause of death for males between 10 and 24 years of age, followed by self-harm (7·8% [5·9–9·3]); whereas self-harm, RTI, and HIV/AIDS were the leading causes of death for females aged 15–19 years (24·9%), and self-harm, road injuries, and tuberculosis were the leading causes of death for ages 20–24 years (21·9%) [5]. A significant proportion (19%) of the global injury related deaths are occurring in the Eastern Mediterranean Region which includes Pakistan [6] [7].

Pakistan has a population of 216.6 million of which 19.4% are youth aged 15–25 years (12). Pakistan is one of the countries with a high number of youth [8]. The 1^st^ National Injury Survey of Pakistan (NISP), which is based on a survey done in 1997, provides the most recent incidences of unintentional injuries from Pakistan. The incidence of all unintentional injuries according to the NISP, in the 16–45 years age bracket, was 45.6 per 1000 per year, while the incidence for road traffic injuries was 15 per 1000 per year (CI: 13.7–16.5). NISP points out the highest incidence of road traffic injuries among laborers at 119.5 per 1000 population, vendors at 106 per 1000 population, businesspersons at 51.9 per 1000, and students at 47.5 per 1000. NISP also identified male sex, age group 16–45 years, and professional laborers/vendors at high risk of road traffic injuries [9]. Another national survey, the 1990–94 National Health Survey of Pakistan (NHSP) [10] reported the annual incidence of all unintentional injuries for the age group from 15–30 years in Pakistan as 46 injuries per 1000 population per year[10]. Furthermore, the annual incidences reported for other causes include: falls at 22.2 per 1000, road traffic injuries at 17 per 1000, poisonings at 3.3 per 1000, and burns at 1.7 per 1000 [10].

Occupational injuries (OIs), although not officially categorized as unintentional injuries, are an important cause of disability and death among youth, as per the Centers for Disease Control and Prevention (CDC) [11]. A higher prevalence of intentional injuries (29%) has been previously reported among youth from vocational schools compared to high school students (17%) [12]. Occupational injuries (also known as work injuries (WIs)), which are injuries that occur at work or in relation to one’s occupation, have been reported to be two times higher among young workers (16–19 years) compared to workers of older ages [13]. Although dedicated studies on OI in vocational youth from Pakistan are rare, one regional study [14] on OIs seen at an emergency department reported higher OIs (47%) among the younger workers (14–18 years) as compared to older workers(18–35 year) 25% from the Rawalpindi region [14]. The majority experienced hand injuries, with fingers being the most common body part affected (67.5%) while (62.5%) also had fractured bones associated with their hand injuries. Higher OIs (82%) were seen in males compared to females (17%) which is a trend also reflected in international literature [14]

Approximately one in ten (i.e., 446 000) youth are engaged in secondary vocational learning in Pakistan, with the majority being male (67%) learners [15]. Previous approaches to health surveys of youth have mainly focused on school-based youth as seen in the CDC’s Global School-based Student Surveys (CDC-GSBHS). School-based youth are generally younger (10–17-year age) and does not represent the older youth (18–24 years) that are non-school going. This is a problem, especially in LMICs where a large proportion of youth are not enrolled in schools [5]. Youth at vocational training institutes (VTIs) is a neglected group that is seldom studied [16–19]. We aimed to determine the incidence, characteristics, and risk factors of unintentional injuries among select youth enrolled at the VTIs in Peshawar, Pakistan.

## Methods

STROBE checklist for cross-sectional studies was used for reporting of this manuscript [20].

### Study design:

This study was conducted on a cross-sectional design. It was part of an explanatory sequential mixed methods study with an overall aim of determining the type, characteristics, and risk factors of unintentional injuries in the youth community. The findings in this manuscript are from the initial quantitative phase of the study.

### Study Setting:

The study was conducted among students of four vocational centers (two male and two female) located in urban areas of Peshawar, Pakistan between February 2022 to September 2022.

### Participants:

Youth at VTIs are a sub-population of youth who are at risk of unintentional injuries. There is a network of four government-run VTIs across Peshawar, Pakistan. These VTIs prepare students for various vocations and skilled jobs. The youth population at VTIs is unexplored in terms of their demographics and behavioral risks for all unintentional injuries.

#### Inclusion criteria

Youth were included in the study if

1. Enrolled in one of the selected VTI in Peshawar over the last 12 months.
2. Between the age of 15–24 years.
3. Able to provide written consent to participate in the study or able to provide written assent and parental consent if less than 18 years.
4. Able to read and write Urdu to fill out the Urdu paper questionnaire. (Pashto is a local spoken language and the majority cannot read or write Pashto, 100% of participants read and wrote Urdu).

#### Exclusion criteria:

Participants were excluded if

1. Less than 18 years or their parents requested withdrawal.
2. Absent on the day(s) of the data collection. (A second attempt was made at recruitment)

### Operational definitions:

An unintentional injury was defined as any injury which was unintentional for which medical treatment was received (at a hospital or a clinic or first aid from his/her mates, teachers, or parents) or it was not treated but caused the injured to miss a half day or more of training or regular activities.

The International Classification of Disease ICD-10 classification for non-fatal unintentional injuries has been used, for example road traffic injuries were defined as body tissue damage resulting from an automobile, motorcycle, pedal cycle or other vehicles, similarly, a fall was defined as body damage resulting from a sudden downward movement due to slipping or tripping such that a person comes to rest on the floor or ground [2].

### Data Collection Instrument:

The WHO Community Survey Guide for Injuries and Violence was used to collect data on unintentional injuries. General sociodemographic information of the youth and injury-specific information were gathered which include the type of injury, mechanism of injury, site of injury, treatment received for injury, place of injury, and demographic characteristics of the youth. Pilot testing of the questionnaire was conducted on 50 participants based on which questions related to counts of injuries were added and a final 81 question-based questionnaire was used for final data collection. Those 50 participants were excluded from the final analysis.

### Variables

#### Age:

Participants with age 15–24 years were included in the study to prevent confounding due to age.

#### Education:

Information was gathered about mother and father education based on the educational system in Pakistan which included middle school, secondary school, higher secondary school, bachelors, master’s and other for uneducated. The categorical variable was then converted into a binary variable as educated and not educated.

#### Family Income:

Income information was gathered as per monthly family income and monthly youth income. A composite family income score was generated by adding the two incomes.

#### Total expected Injuries count:

The total injuries count was calculated by adding up the individual injuries counts. Each injury was taken as a mutually exclusive event in previous 12 months. Information was gathered about individual unintentional injuries count including road traffic injuries, falls, burns, drownings, poisonings, in-training injuries, and hand injuries. ICD-10 coding was used for reporting unintentional injuries.

#### Bias:

The age restriction was done to prevent confounding due to age. Adjustment was done for sex with females taken as reference except for burns where female injuries were reported higher compared to males. Confounding was checked. Participants were particularly asked to report counts of their injuries in the last 12 months to prevent recall bias. Verification was done for recall bias on a group of 20 participants who filled the questionnaire twice.

#### Study size:

Quantitative sample: 550 (15–24-year-old) youth enrolled at VTIs in Peshawar over the last 12 months were sampled. (Assuming an incidence of unintentional injuries among 16–45 years age as (45.6/1000) 0.05% [9], a (0.03) 3% risk difference of unintentional injuries between males and females [21], using a two sided test at a confidence level of 95% and a power of 80% a minimum sample of was calculated using the formula. A non-response rate of 10% (50) was added to achieve a total sample of 550). A convenient sampling strategy was used

Ethical approval was obtained from the Ethical Review Committee at Aga Khan University. This survey was part of a mixed methods study with an initial quantitative survey followed by qualitative interviews. Administrative approval was obtained from the Directorate of Technical & Vocational Education Training Authority TVETA Peshawar before the commencement of data collection and dispersion of consent forms. Ethical approval of the project was obtained in February 2022.

#### Statistical methods:

[22]. Missing data were eliminated which reduced the sample size from 649 to 547. X Descriptive statistics were reported as frequency and percentages. Multivariable Negative Binomial Regression (NBREG) analysis was applied, and adjusted incidence rate ratios were calculated for age and sex.

## Results

A total 547 students participated in the study out of 191(35%) were female and 356(65%) participants were males. The majority of youth were unmarried 502(92%) were educated while a small percentage were uneducated. Maternal education reported by participants was lower at 326(60%), compared to paternal education at 437(80%). A median monthly income of 35000 PKR per family was reported, 239(44%) youth reported they lived in crowded families (>8 family members) as in table 1.

Among the participants there were very few smokers 15(2.7%), the majority being nonsmokers 532(97.3%). Substance use was self-reported by 9(2%) of the participants. A small number of 43(8%) participants reported that they had carried a gun in the previous 30 days for personal protection. 50% of the youth reported the use of a helmet while 240(43.9%) reported seat belt use. as shown in table 2.

Irrespective of sex the RTIs 197(39%) were the most common injuries reported, as shown in table 3. Most RTI incurred were as a driver (26%) as shown in Table 4, while passenger and pedestrian injuries reported were 14.08% and 12.07% respectively (Table 4). RTIs were followed by burns 89(18%) and falls 79(16%) while drownings 28(5%) and poisonings 15(3%) were the least common non-fatal injuries reported by youth (table3). 287(52%) of youth did not receive any first aid services, further details regarding first aid utilization are provided in table 5.

Participants were asked if they had any unintentional injuries in the previous 12 months. The incidence rate reported corresponds to the year 2021–22. Among all unintentional injuries reported the RTIs were the highest197(39%) (table 3). When comparing male to females, RTIs had the highest incidence rate 3.24[2.35–5.3] in males (table 6). Similarly, males also had a higher incidence of falls 1.30 [0.74–2.27], poisonings 2.14 [0.57–7.58], drownings 2.46(0.84–7.21) and in-training occupational injuries 1.28 [1.19–3.74]. On the contrary, females had a highest incidence rate of burns 2.19 [1.785–3.46] as compared to males (table 6).

The association of sociodemographic risk factors is shown in table 7. It can be noted that the incidence rate of total unintentional injuries was 4.05 times high in males compared to females (<0.001) after adjusting for age. No significant difference was noted in the incidence rate for youth in age ≤ 19 years when compared to youth with age > 19 years. Education of self and father were found to be significant risk factors for unintentional injuries. Youth lacking self-education had an incidence rate of 4.6 [1.12–18.91] (*p*=0.034) as compared to educated youth. Youth lacking fathers’ education had an incidence rate of 4.71 [2.12–10.49] (p<0.001) for unintentional injuries compared to youth whose fathers had education. Mothers’ education did not show a significant impact of the unintentional injuries. Youth with low-income status (family income 35000 PKR or less) had 2.04 [1.04–4.02] (*p*=0.039*) higher incidence of injuries compared to youth with high income status (family income 35000 PKR). Youth that reported carrying a gun for protection when leaving home in the last 30 days had 6.59 [2.54–17.11] times higher incidence rate (p<0.001). Other significant risk factors noted were lack of helmet use 4.54 [2.12–9.76] (*p*=<0.001), lack of seat belt use 1.3 [1.14–1.69] (*p*= <0.001), smoking 1.25 [1.05–2.69] (*p*=0.049) living in crowded families (family >=8 members) 3.59 [3.11–5.07] (*p*= <0.001).

## Discussion

In our study we determined the current trends in unintentional injuries among youth from Peshawar Pakistan. This is a first study to our knowledge to determine the in-training occupational injuries along with other unintentional injuries in youth population from Peshawar Pakistan. Our study shows that RTIs remain the top non-fatal unintentional injury in youth irrespective of age and sex. Burns and falls were second and third most common injuries reported after RTIs. Sex was associated with all unintentional injuries, with males having 1–7 times higher incidence of unintentional injuries except for burns which were higher in females compared to males. Occupational injuries were noted to be high. A sex association was noted in occupational injuries as well. The findings of (NHSP 1990–94) [10] a national level survey in Pakistan, shows a high percentage of fall injuries (48%), followed by road traffic injuries 37.6%, while poisonings (7%) and burns (3) were lower in percentages [10]. The current pattern in our study is different from the trend noted in NHSP. Youth reported RTIs were the number one cause of injury in our study while burn and falls were second and third most common cause of injury reported along with occupational injuries. The reason for this shift could be that NHSP survey was done 30 years ago and since then a global shift towards higher RTIs has been noted among youth generally.

The NISP [9] which was another national level injury survey done in Pakistan in 2004 reported a relative risk of RTI as 2.48 times (16–45-year age) and 3.24 in males compared to female, while a relative risk of 1.63 for all other injuries was reported. Our findings are like NISP; our incidence rate of RTIs in males is 3.53 as compared to females which is higher. In a 2021 trauma registry [23] which was made by the Lady Ready Hospital in Peshawar, prospective data were collected in the hospital for a total of 267 trauma patients from Peshawar, it was noted that Motor Vehicle Collison (MVC) was the most common cause of injury 122 (46%). Pedestrians were the most injured (45%), followed by passengers (31%) and drivers (24%). While in our study driver injuries were higher at 48% while passengers (28%) and pedestrians (24%) injuries were lower. This is contrary to the study by Tanoli et [23]. A study published by Shah et al [24] who obtained data from 30 police stations in Peshawar between 2015–16 and analyzed the mode of transport for the RTI, results showed that 74% of fatal and 59% of the non-fatal crashes in Peshawar were pedestrians [24].

Helmet use is a key determinant in decreasing head injuries in RTIs. A study done on crash injuries in children 18 years and younger who rode bicycles and ATVs reported that residence in a county with both lower median income and scholastic graduation rates associated were associated with unhelmeted crashes, and lower median income significantly predicted unhelmeted crashes. This study revealed socioeconomic factors that identify communities with greatest need for injury prevention initiatives [25]. A study done in Bangladesh by Tana et al found a 1.17 times (95% CI 1.02—1.35; p 0.03) higher risk of head injuries in road traffic accidents, in the unhelmeted group compared to the helmeted group [26].

India being a neighboring South Asian country has demographic similarities with Pakistan. An epidemiologic survey done on the adolescent population from India, by Reddy et al in 2021 [27], showed that 73% of the adolescents sampled had unintentional injuries [27], this is similar to our study where 503 injuries were reported which is approximately I injury per participant (*N*=547). Reddy et al reported male sex as a statistically insignificant risk factor with OR of 1.0, this is contrary to our study where males had 1.6 times higher incidence for total unintentional injuries, with a statistical significance. Another important risk factor identified by Reddy et al [22] was smoking though insignificant statistically, on the contrary in our study smokers had a 12.8 higher incidence of total unintentional injuries compared to non-smoker (*P* <0.001) when adjusted for age and sex. Other risk factors identified by Reddy et al [22] were overcrowding [27]. This is similar to our study, which showed that youth with > 8 family members had higher incidence of total expected injuries when adjusted for age and sex (*P*=0.002).

The economic situation of a family has shown to impact the injuries, in our study youth with a family income less than 35000 PKR per month had a significantly higher incidence for total unintentional injuries compared to youth with > 35000 PKR family income. In the GBD 2015 study [28] a factor known as “the sociodemographic index” (SDI) was introduced to better understand the sociodemographic risk factors for unintentional injuries. The SDI was created by combining the income, education, and fertility for various countries. A decreasing trend for injuries was noted with increasing SDI which means when the socioeconomic status of the youth increased the burden of injuries decreased. However, it was also reported to be a multifactorial phenomenon [28]. RTIs showed an increasing trend at low and middle SDI but declined at a high SDI. A 10% increase in GDP in a lower-income country (GDP/Capita <1600) is expected to raise the number of road crashes by 7.9%, the number of traffic injuries by 4.7%, and the number of deaths by 3.1% through a mechanism that is independent of population size, vehicle counts, oil use, and roadway availability. Increases in GDP in richer countries appear to reduce the number of traffic deaths, but do not reduce the number of crashes or injuries, all else equal. Greater petrol use and alcohol use are related to more traffic fatalities in rich countries, all else equal[29].

Near drownings reported from India by Reddy et all [27] were 0.6%, falls 36% and burns 19.4%, our findings are different, our percentages for higher burns than falls. Mortality due to unintentional poisoning in LMICs is four times (2/100,000) as compared to higher high-income countries (HICs) (0.5/100,000). Pakistan National Emergency Department Surveillance for unintentional injuries conducted across seven major emergency departments (2010–11) reported a 10% (25/211) incidence of unintentional poisonings among 0–15-year-old children. Males were affected at a higher rate (68%) compared to females (31.8%) [30]. Mortality due to unintentional drowning is the highest among children, especially males in low-SDI to middle-SDI countries. China, India, Pakistan, and Bangladesh accounted for 51.2% of all drowning deaths in 2017 [31]. Our findings confirm a high incidence rate of the drowning of 2.46 times higher in males compared to females.

When compared to the findings from youth in Brazil the prevalence of self-reported occupational accidents was 55% [32]. Data from the New Jersey Department of Education (NJDOE) on vocational youth from December 1998–June 2015 reported that males had (72%) while females had (28%) occupational injuries, these injuries were mild and mostly involved the hand’s [33]

## Conclusion

Our study confirms RTI as the common injury among vocational youth followed by burns and falls while poisonings and drownings were the least reported injuries by youth.

Occupational injuries that occurred during the vocational training were high at 95 (18%). Females had a higher incidence rate of burns compared to males, while males had a higher incidence of RTIs, falls, poisonings, and drownings. Continued efforts towards decreasing RTI, burns, falls, and occupational injuries among youth are needed. Youth interventions in Pakistan should focus on youth behavior like carrying a gun, smoking, helmet use, seat belt use, and literacy. Other targeted interventions could focus on families with low socioeconomic status, crowded living situations, and low paternal education. Specific injury prevention programs are needed for preventing injuries in vocational youth.

### Strengths and Limitations:

This study gathered data on self-reported non-fatal unintentional injuries which are seldom reported. The study is specific to the youth population. Data were gathered in the community provides a good picture of the often-missed non-fatal community unintentional injuries. Multiple types of unintentional injuries were recorded in the survey along with occupational injuries. Data on unintentional injuries were gathered as counts over the previous 12 months and reported as IRRs. Lack of follow-up of participants, cross-sectional methodology, and fewer female participants compared to males were limitations of the study. Age and sex are the biggest confounders which were addressed through restriction and segregation at both the design and analysis stages.. Restriction of the age group to 16–24 years was done to minimize confounding due to age, and adjustment was also done at analysis stage for age and sex to further control for confounding.

## Availability of Data and materials:

The datasets used in this study are available from the corresponding author on reasonable request.

## Figures and Tables

**Table 1:** Sociodemographic characteristics of students

Descriptive and Demographics Results	Total
**N**	**547**
**Age in years**	
**Median (IQR)**	19 (18 −20)
**Age Range (Max - Min)**	**(24 −16)**
**Sex**	
Male	356(65%)
Female	191(35%)
**Marital Status**	
Married	45(8%)
Unmarried	502(92%)
**Center**	
GTVCBgulbahar	54(9.8%)
GTVCBhayatabad	321 (58.6%)
GTVCWgulbahar	12(2.1%)
GTVCWhayatabad	160(29.2%)
**Self Edcuation**	
Non-Educated	12(2.1%)
Educated	535(97.8)
**Mother Education**	
Non-Educated	221 (40%)
Educated	326(60%)
**Father Education**	
Non-Educated	110(20%)
Educated	437(80%)
**Monthly Income (PKR)**	
Median [IQR]	30000 (40000 – 22000)
**Monthly Income (PKR) Classification**	
<=35000 PKR	272(50%)
>35000 PKR	275(50%)
**Family Members in Household**	8.4 ±3.8
Range (Max - Min)	(29 −1)
**Family Members Classification**	
<=8 members	308(56%)
>8 members	239(44%)

**Table 2:** Behavioral risk factors of youth participants (N=547)

Descriptive and Demographics Results	Total N=547 (%)
**Helmet Use**	
No USE	273(50%)
Used	274(50%)
**Smoking Status**	
No Smoker	532(97.3%)
Smoker	15(2.7%)
**Substance Use**	
No USE	538(98%)
Used	9(2%)
**Gun Used**	
No USE	504(92%)
Used	43(8%)
**Seat Belt Use**	
No USE	307(56.1%)
Used	240(43.9%)

**Table 3:** Types of unintentional injuries along with International Classification of Disease-10 code.

Road Traffic Injuries (C.1)	197(39%)
Burns (C.2.3)	89(18%)
Falls (C.2.1)	79(16%)
In-training occupational Injuries	95(18%)
Drowning Category (C.2.2)	28(5%)
Poisons Category (C.2.4)	15(3%)
Total	N=503

**Table 4:** Role of an injured participant in RTI and type of vehicles causing injury.

**Type of vehicle in recent RTI**	
walking	47 (8.59%)
nonmotorized vehicle	5 (0.91%)
bicycle	16 (2.93%)
motorcycle	164 (29.98%)
car	33 (6.03%)
pickup van jeep or minibus vehicle that seats less than 10 people	8 (1.46%)
bus	4 (1 %)
three-wheel motorized vehicle	6 (1.10%)
Other	264(58%)
**Role in recent RTI**	
pedestrian	66(12.07%)
driver	142(26%)
passenger	77(14.08%)
Other	261 (48%)
**Agent in recent RTI**	
pedestrian	26(4.75%)
bicycle	12(2.19%)
motorcycle	70(12.80%)
motorized vehicle	115(21.02%)
fixed object	48(8.78%)
Other	276(50%)

**Table 5:** First aid and healthcare utilization for the unintentional injuries

**Was First aid received?**	
Yes	188(34%)
No	287(52%)
Other	72(14%)
**Which medical service was availed?**	
Hospital	195(35%)
Clinic	54(10%)
Healthcare center	4(1%)
General Medical Practitioner	23(4%)
Community worker/traditional	5(1%)
Other	266(49%)
**Was admitted to the hospital?**	
No	478(87%)
Yes	68(13%)
**No of days admitted in the hospital**	Mean=0.12, Sd=0.33, range 0–1

**Table 6:** Incidence rate ratios of total self-reported unintentional injuries among youth for year 2021–22 with adjustment for sex and age.

Injury types		
	Incidence Rate Ratio IRR [95% Confidence Interval]	
	Male (adjusted)	Age (Adjusted)
Total Injury	1.63 [1.31–2.04]	0.93 [0.88–0.99]
RTI	3.24[2.35–5.3]	0.95 [0.87–1.04]
Poisoning	2.14 [0.57–7.58]	0.85 [0.61–1.16]
Falls	1.30 [0.74–2.27]	0.82 [0.70–0.96]
Burns (female)	2.19 [1.785–3.46]	0.85 [0.77–0.94]
Drowning	2.46(0.84–7.21)	0.89 [0.75–1.05]
Occupational Injuries	1.28 [1.19–3.74]	1.02 [0.89–1.16]

**Table 7:** Association of sociodemographic and behavioral risk factors with total unintentional injuries in youth, adjusted for sex and age.

Factors	Univariate		Multivariate	
	RR [95% CI]	P-value	RR [95% CI]	P-value
Male gender	3.29 [1.57 −6.92]	0.002*	4.05 [1.84 −8.91]	<0.001*
Age ≤19 years	2.05 [1 −4.2]	0.049*	1.61 [0.8 −3.27]	0.184
Marital Status (Married)	0.6 [0.17 −2.08]	0.421	-	-
Helmet Use (No)	2.12 [1 −4.49]	0.049*	4.54 [2.12 −9.76]	<0.001*
Smoker (yes)	1.29 [1.06 −3.35]	0.023*	1.25 [1.05 −2.69]	0.049*
Gun Use (Yes)	1.16 [1.06 −1.48]	<0.001*	6.59 [2.54 −17.11]	<0.001*
No Seat Belt Used	1.34 [0.6 −0.6]	<0.001*	1.3 [1.14 −1.69]	<0.001*
Self-Education (Non-Educated)	5 [1.15 −21.85]	0.032*	4.6 [1.12 −18.91]	0.034*
Father Education (Non-Educated)	3.06 [1.43 −6.55]	0.004*	4.71 [2.12 −10.49]	<0.001*
Mother Education (Non-Educated)	0.91 [0.44 −1.86]	0.794	-	-
Family Income (<=35000 PKR)	2.05 [1.01 −4.16]	0.046*	2.04 [1.04 −4.02]	0.039*
Family (>=8 members)	2.37 [1.13 −3.67]	0.009*	3.59 [3.11 −5.07]	<0.001*

IRR: Incidence Rate Ratio, (adjusted multivariable IRR)

Negative Binomial Distribution,

Dependent variable: Total Injury Counts.

95% C.I: Confidence interval

## Abbreviations

AKU: Aga Khan University
CDC: Center of Disease and Control
EMR: Eastern Mediterranean Region
ERC: Ethical Review Committee
GTVCW-G: Government Technical and Vocational Center Women-Gulbahar
GTVCB-G: Government Technical and Vocational Center Boys-Gulbahar
GTVCW-H: Government Technical and Vocational Center Women-Hayatabad
GTVCB-H: Government Technical and Vocational Center Boys-Hayatabad
JHU-Af-Pak ICTIRT: Johns Hopkins-Afghanistan-Pakistan International Collaborative Trauma and Injury Research Training program at Aga Khan University Emergency Department
NCD: Non-Communicable disease
QUAL: Qualitative
quan: Quantitative
Stata Corp 15.1: Statistical Analysis Software Stata Corp LLC version 15.1
STROBE: Strengthening the Reporting of Observational studies in Epidemiology
ICD-10: International Statistical Classification of Diseases and Related Health Problems, tenth revision.
UNESCO: United Nations Educational, Scientific and Cultural Organization
UNEVOC: UNEsco and VOCation
UI: Unintentional Injuries
TVET: Technical Vocational Education and training
VTI: Vocational Training Institute
YRBSS: Youth Risk Behavior Surveillance System
WHO: World Health Organization
WI: Work Injuries
